# The distribution of acquired peripheral nerve injuries associated with severe COVID-19 implicate a mechanism of entrapment neuropathy: a multicenter case series and clinical feasibility study of a wearable, wireless pressure sensor

**DOI:** 10.1186/s12984-022-01089-1

**Published:** 2022-10-08

**Authors:** Colin K. Franz, Nikhil K. Murthy, George R. Malik, Jean W. Kwak, Dom D’Andrea, Alexis R. Wolfe, Ellen Farr, Melanie A. Stearns, Swati Deshmukh, Jinny O. Tavee, Fang Sun, Kevin N. Swong, Leslie Rydberg, R. James Cotton, Lisa F. Wolfe, James M. Walter, John M. Coleman, John A. Rogers

**Affiliations:** 1 Shirley Ryan Ability Lab, 26-North, 355 E. Erie Street, Chicago, IL 60611 USA; 2 Department of Physical Medicine and Rehabilitation, Northwestern University Feinberg School of Medicine, Chicago, IL USA; 3 The Ken and Ruth Davee Department of Neurology, Northwestern University Feinberg School of Medicine, Chicago, IL USA; 4 Querrey Simpson Institute for Bioelectronics, Northwestern University, Evanston, IL USA; 5 McGaw Medical Center, Northwestern University Feinberg School of Medicine, Chicago, IL USA; 6 Department of Neurological Surgery, Northwestern University Feinberg School of Medicine, Chicago, IL USA; 7 Department of Physiatry, Hospital for Special Surgery, New York, NY USA; 8 Department of Mechanical Engineering, Northwestern University, Evanston, IL USA; 9 The Division of Pulmonary and Critical Care, Department of Medicine, Northwestern University Feinberg School of Medicine, Chicago, IL USA; 10 Department of Physical Medicine and Rehabilitation, Mayo Clinic College of Medicine and Science, Rochester, MN USA; 11 Marianjoy Rehabilitation Hospital, Northwestern Medicine, Wheaton, IL USA; 12 Department of Radiology, Beth Israel Deaconess Medical Center, Boston, MA USA; 13 Division of Neurology & Behavioral Health, National Jewish Health, Denver, CO USA; 14 Department of Materials Science and Engineering, Northwestern University, Evanston, IL USA; 15 Department of Biomedical Engineering, Northwestern University, Evanston, IL USA; 16 Department of Chemistry, Northwestern University, Evanston, IL USA; 17 Department of Electrical and Computer Engineering, Northwestern University, Evanston, IL USA

**Keywords:** Rehabilitation, Neuromuscular, COVID-19, Peripheral nerve injury, Neuropathy, Brachial plexus, Wearable sensor, Intensive care unit

## Abstract

We diagnosed 66 peripheral nerve injuries in 34 patients who survived severe coronavirus disease 2019 (COVID-19). We combine this new data with published case series re-analyzed here (117 nerve injuries; 58 patients) to provide a comprehensive accounting of lesion sites. The most common are ulnar (25.1%), common fibular (15.8%), sciatic (13.1%), median (9.8%), brachial plexus (8.7%) and radial (8.2%) nerves at sites known to be vulnerable to mechanical loading. Protection of peripheral nerves should be prioritized in the care of COVID-19 patients. To this end, we report proof of concept data of the feasibility for a wearable, wireless pressure sensor to provide real time monitoring in the intensive care unit setting.

## Introduction

Severe coronavirus disease 2019 (COVID-19) frequently requires intensive care unit (ICU) admission and prolonged periods of mechanical ventilation. ICU acquired weakness (ICU-AW) is an established neuromuscular complication of severe COVID-19 [1] since between 5 and 17% of patients develop a critical illness from severe acute respiratory syndrome coronavirus 2 infection [2–4]. ICU-AW clinically manifests as a myopathy, polyneuropathy, or a combination of both [5]. Regardless, all ICU-AW phenotypes produce diffuse, symmetric symptoms, so if asymmetric neuromuscular symptoms are noted this should trigger further scrutiny for a superimposed process, such as a focal peripheral nerve injury (PNIs) [6, 7]. PNIs may be superimposed on ICU-AW and easily missed in the acute care setting without a detailed neuromuscular assessment. Malik et al. described a case series of COVID-19 PNIs in 12 patients admitted to an inpatient rehabilitation [8], which has subsequently been reaffirmed by a series of other published case series in survivors of severe COVID-19 [9–13]. The anatomical distribution of PNIs implies a role for mechanical forces as these localizations mirror sites known to be vulnerable to compression and/or traction injury. [6, 7]

**Fig. 1:**
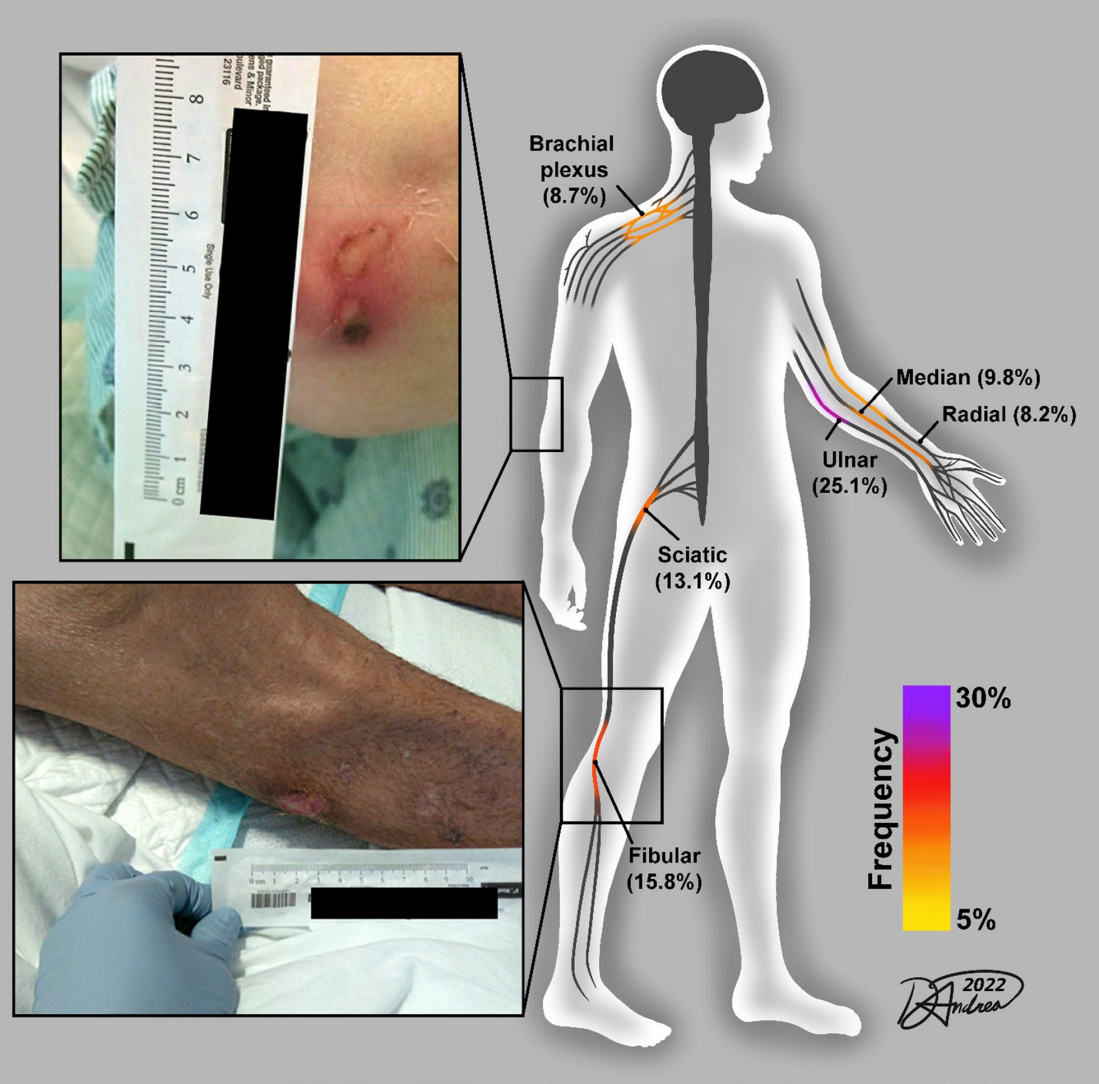
Graphical summary of common nerve injury sites in survivors of COVID-19. This summary includes current data and several recent case series of peripheral nerve injuries associated with COVID-19. Inset photographs show examples skin pressure sores overlaying the nerve compression sites – elbow (top) and fibular head (bottom)

Unfortunately, the recovery from PNI is notoriously slow and frequently incomplete in the general population [14], and the demographics of severe COVID-19 patients include enrichment of known risk factors for worse outcomes after PNI such as advanced age, obesity and diabetes mellitus [8]. There is little doubt that acquired PNIs contribute to long term disability in survivors of severe COVID-19 with reported incidences between 14.5 and 16% [8, 9]. Here we use a multicenter, retrospective chart review to define the types and distribution of PNIs in survivors of severe COVID-19, which is integrated with several smaller published case series [8–13]. Additionally, we provide feasibility data on a wearable, wireless pressure sensor to provide real time monitoring at the elbow, which is inspired by the clinical observation that ulnar neuropathy at the elbow is the single most common nerve compression site in hospitalized COVID-19 survivors (Fig. 1).

## Methods

Study approval was granted by the Northwestern University Institutional Review Board. Patients were identified in retrospective fashion for admissions between April 1, 2020 and March 31, 2021 to 1 of 3 academically affiliated medical centers. This included 2 inpatient rehabilitation hospitals (Shirley Ryan AbilityLab, Chicago, IL, USA; Marianjoy Rehabilitation Hospital, Wheaton, IL, USA) and 1 tertiary care hospital (Northwestern Memorial Hospital, Chicago, IL, USA). For the majority of PNIs (62 out of 66), the diagnosis and localization were supported by electrodiagnostic testing and/or neuromuscular ultrasound (Table 1). We also performed a literature review and included other case series we found (minimum ≥ 5 patients; Table 2). Due to the retrospective nature of this study, public and patient involvement in this research study design was not obtained. Also, we excluded 12 cases (21 PNIs) that were previously reported [8] from our primary analysis (Table 1) but did include them in our literature review. Unless otherwise noted, values are expressed as mean ± standard deviation.

**Table 1:** Summary of 34 new cases of peripheral nerve injury after severe COVID-19

#	Age/Sex	Site	Risk Factors	PNI sites (laterality)	Diagnostics	Clinical presentation(s)
1	60s/M	SRAlab	DM, Obese	Sciatic (left), Fibular (right)	EDX (axonal)	Bilateral foot drop; Left worse than right
2	50s/M	SRAlab	DM	Ulnar (left)	EDX (demyelin.)	Left hand weakness
3	40s/F	SRAlab	Obese	Cervical radiculopathy (left)	EDX (axonal)	Neck pain; Left shoulder and elbow flexion weakness
4	60s/M	SRAlab	DM	Femoral (left), Obturator (left), Spinal accessory (right)	EDX (axonal), MRI (pelvis)	Left thigh pain and paresthesia; Left knee extension weakness; Right sided neck and shoulder pain
5	70s/M	SRAlab	None	Sciatic (right), Fibular (left)	EDX (axonal)	Bilateral foot drop; Right worse than left
6	30s/M	SRAlab	Obese	Sciatic (right), Fibular (left), Phrenic (right)	EDX (axonal), US (phrenic)	Bilateral foot drop; Right worse than left; Shortness of breath on exertion
7	40s/M	SRAlab	None	Phrenic (right)	US (phrenic)	Shortness of breath on exertion
8	60s/M	SRAlab	HIV	Radial (right), Ulnar (right), Median (right), Sciatic (right), Ulnar (left), Median (left)	EDX (axonal; but demyelin. median)	Diffuse, but asymmetric weakness and sensory disturbance
9	60s/M	SRAlab	Obese	Brachial plexopathy (right), Ulnar (right), Median (right), Phrenic (right)	EDX (axonal; but demyelin. ulnar), US (phrenic)	Diffuse right arm weakness and sensory disturbance; Shortness of breath
10	60s/M	SRAlab	None	Fibular (right)	EDX (axonal)	Right foot drop; Paresthesia in right foot
11	70s/M	SRAlab	CKD	Femoral (right), Obturator (right), Fibular (right)	EDX (axonal), MRI (pelvis)	Right knee buckling and weakness; Right foot drop
12	50s/M	SRAlab	DM	Cervical radiculopathy (left), Ulnar (left), Phrenic (right)	EDX (axonal), MRI (neck/shoulder), US (phrenic)	Left shoulder and elbow flexion weakness; Left hand weakness; Shortness of breath on exertion
13	60s/M	SRAlab	DM	Sciatic (right), Phrenic (right)	EDX (axonal), US (phrenic)	Right foot drop; Shortness of breath on exertion
14	30s/M	SRAlab	Obesity	Sciatic (left)	EDX (axonal)	Left foot drop
15	40s/M	SRAlab	None	Fibular (left), Fibular (right)	EDX (axonal)	Bilateral foot drop
16	60s/M	SRAlab	None	Ulnar (right), Radial (right), Median (right), Ulnar (left), Radial (left)	EDX (axonal)	Bilateral forearm and hand weakness in extension and flexion; Worse on right
17	70s/F	SRAlab	DM	Sciatic (left)	EDX (axonal)	Left foot drop
18	70s/M	SRAlab	DM, CKD	Fibular (left)	EDX (axonal)	Left foot drop
19	50s/M	SRAlab	Obese	Sciatic (right), Fibular (left)	EDX (axonal)	Bilateral foot drop; Worse on right side
20	40s/M	SRAlab	DM, Obese	Fibular (right), Fibular (left)	EDX (axonal)	Bilateral foot drop
21	70s/M	SRAlab	None	Ulnar (left), Lateral femoral cutaneous (left)	EDX (axonal, but clinical LFCN)	Left hand weakness; Painful paresthesia in left anterolateral thigh
22	50s/M	SRAlab	None	Ulnar (left)	EDX (axonal)	Left hand weakness
23	50s/M	NMH	DM, Obese	Radial (right)	None (clinical)	Right wrist drop and forearm pain
24	30s/F	NMH	DM, Obese	Sciatic (left)	EDX (axonal)	Left foot drop
25	30s/M	NMH	Obese	Ulnar (left)	EDX (axonal)	Left hand weakness
26	40s/M	NMH	Obese	Radial (left)	EDX (axonal)	Left wrist drop
27	20s/M	NMH	DM, Obese	Ulnar (left)	EDX (axonal)	Left hand weakness
28	20s/M	NMH	Obese	Brachial plexopathy (left), Radial (right)	EDX (axonal)	Left shoulder and elbow flexion weakness; Right wrist drop
29	60s/M	NMH	Obese	Brachial plexopathy (left), Ulnar (left), Sciatic (left), Fibular (right)	EDX (axonal; but demyelin. ulnar)	Left shoulder and elbow flexion weakness; Left hand weakness; Bilateral foot drop
30	30s/F	MRH	DM, Obese	Fibular (right), Fibular (left)	EDX (axonal)	Bilateral foot drop
31	60s/M	MRH	None	Fibular (right)	EDX (axonal)	Right foot drop
32	60s/M	MRH	DM, Obese	Brachial plexopathy (right)	EDX (axonal)	Diffuse right arm weakness and numbness
33	70s/M	MRH	DM, Obese	Fibular (right), Fibular (left)	None (clinical)	Bilateral foot drop
34	30s/M	MRH	DM, Obese	Median (right), Median (left)	EDX (axonal)	Bilateral hand weakness and paresthesia

PNI, peripheral nerve injury; SRAlab, Shirley Ryan AbilityLab (rehabilitation hospital); NMH, Northwestern Memorial Hospital; MJH, Marianjoy Rehabilitation Hospital; DM, diabetes mellitus; HIV, human immunodeficiency virus; CKD, chronic kidney disease; EDX, electrodiagnostics; MRI, magnetic resonance imaging; US, ultrasound; demyelin., demyelinating; LFCN, lateral femoral cutaneous nerve. In accordance with journal policy, patient age is expressed according to their decade of life

**Table 2:** Summary of patient characteristics from previously published case series

#	Age/ Sex	PNI sites (laterality)	Citation
1	70 s/M	Radial (left), Ulnar (left), Median (left), Median (right)	Malik et al. [8]
2	60 s/F	Radial (right), Median (right), Ulnar (right)	Malik et al. [8]
3	60 s/M	Brachial plexopathy (right)	Malik et al. [8]
4	70 s/F	Radial (left)	Malik et al. [8]
5	40 s/M	Ulnar (left), Ulnar (right), Musculocutaneous (right), Median (left)	Malik et al. [8]
6	60 s/M	Ulnar (right)	Malik et al. [8]
7	50 s/M	Sciatic (left)	Malik et al. [8]
8	50 s/M	Brachial plexopathy (right)	Malik et al. [8]
9	60 s/M	Ulnar (right), Sciatic (right)	Malik et al. [8]
10	80 s/F	Cervical radiculopathy (left), Fibular (left)	Malik et al. [8]
11	70 s/F	Sciatic (left)	Malik et al. [8]
12	20 s/F	Fibular (left), Lateral femoral cutaneous (right)	Malik et al. [8]
13	N/A	Sciatic (right)	Needham et al. [9]
14	N/A	Ulnar (left), Fibular (right)	Needham et al. [9]
15	N/A	Ulnar (left), Ulnar (right), Fibular (left), Fibular (right)	Needham et al. [9]
16	N/A	Ulnar (right), Radial (right), Sciatic (right), Sciatic (left)	Needham et al. [9]
17	N/A	Musculocutaneous (right), Musculocutaneous (left), Sciatic (right), Sciatic (left)	Needham et al. [9]
18	N/A	Median (right), Median (left), Musculocutaneous (left), Radial (left)	Needham et al. [9]
19	N/A	Fibular (right), Lateral femoral cutaneous (left)	Needham et al. [9]
20	N/A	Ulnar (left), Median (right), Median (left), Axillary (right), Sciatic (left), Sciatic (right)	Needham et al. [9]
21	N/A	Median (right)	Needham et al. [9]
22	N/A	Ulnar (left), Ulnar (right)	Needham et al. [9]
23	N/A	Ulnar (right), Median (left), Lateral femoral cutaneous (right)	Needham et al. [9]
24	60s/M	Brachial plexopathy (left)	Miller et al. [11]
25	40s/F	Median (right), Brachial Plexopathy (left)	Miller et al. [11]
26	60s/M	Brachial plexus (left), Brachial plexus (right), Radial (left), Ulnar (right)	Miller et al. [11]
27	60s/M	Brachial plexus (right)	Miller et al. [11]
28	40s/M	Ulnar (left), Axillary (left)	Miller et al. [11]
29	60s/M	Ulnar (left), Ulnar (right)	Miller et al. [11]
30	50s/M	Ulnar (left), Ulnar (right), Brachial plexus (left)	Miller et al. [11]
31	50s/F	Brachial plexus (right)	Miller et al. [11]
32	50s/M	Ulnar (left), Radial (left)	Miller et al. [11]
33	60s/M	Ulnar (right), Axillary (left)	Miller et al. [11]
34	40s/M	Ulnar (left), Ulnar (right), Brachial Plexus (left), Musculocutaneous (right)	Miller et al. [11]
35	50s/M	Ulnar (left), Musculocutaneous (right)	Miller et al. [11]
36	50s/M	Spinal accessory (left)	Miller et al. [11]
37	30s/M	Ulnar (right), Radial (right)	Miller et al. [11]
38	60s/M	Ulnar (right)	Brugliera et al. [12]
39	40s/M	Ulnar (left), Ulnar (right), Suprascapular (left), Suprascapular (right), Axillary (left), Axillary (right)	Brugliera et al. [12]
40	50s/M	Ulnar (left), Ulnar (right)	Brugliera et al. [12]
41	40s/M	Ulnar (left)	Brugliera et al. [12]
42	50s/M	Brachial plexus (left)	Brugliera et al. [12]
43	60s/M	Brachial plexus (right), Musculocutaneous (right), Lumbosacral plexus (left)	Brugliera et al. [12]
44	70s/M	Ulnar (right), Median (right)	Brugliera et al. [12]
45	40s/M	Ulnar (right)	Brugliera et al. [12]
46	60s/M	Fibular (left)	Chang et al. [10]
47	60s/M	Fibular (left)	Chang et al. [10]
48	50s/F	Fibular (left)	Chang et al. [10]
49	50s/M	Fibular (right)	Chang et al. [10]
50	30s/M	Fibular (right)	Chang et al. [10]
51	70s/M	Femoral (right)	Liu et al. [13]
52	40s/M	Sciatic (right), Sciatic (left)	Liu et al. [13]
53	40s/M	Sciatic (right), Sciatic (left)	Liu et al. [13]
54	70s/F	Lumbar plexus (right)	Liu et al. [13]
55	60s/F	Radial (left)	Liu et al. [13]
56	70s/M	Fibular (left)	Liu et al. [13]
57	60s/M	Fibular (right)	Liu et al. [13]
58	40s/M	Fibular (right)	Liu et al. [13]

*PNI* peripheral nerve injury, *M* male, *F* female, *N/A* not available. In accordance with journal policy, patient age is expressed according to their decade of life

**Fig. 2:**
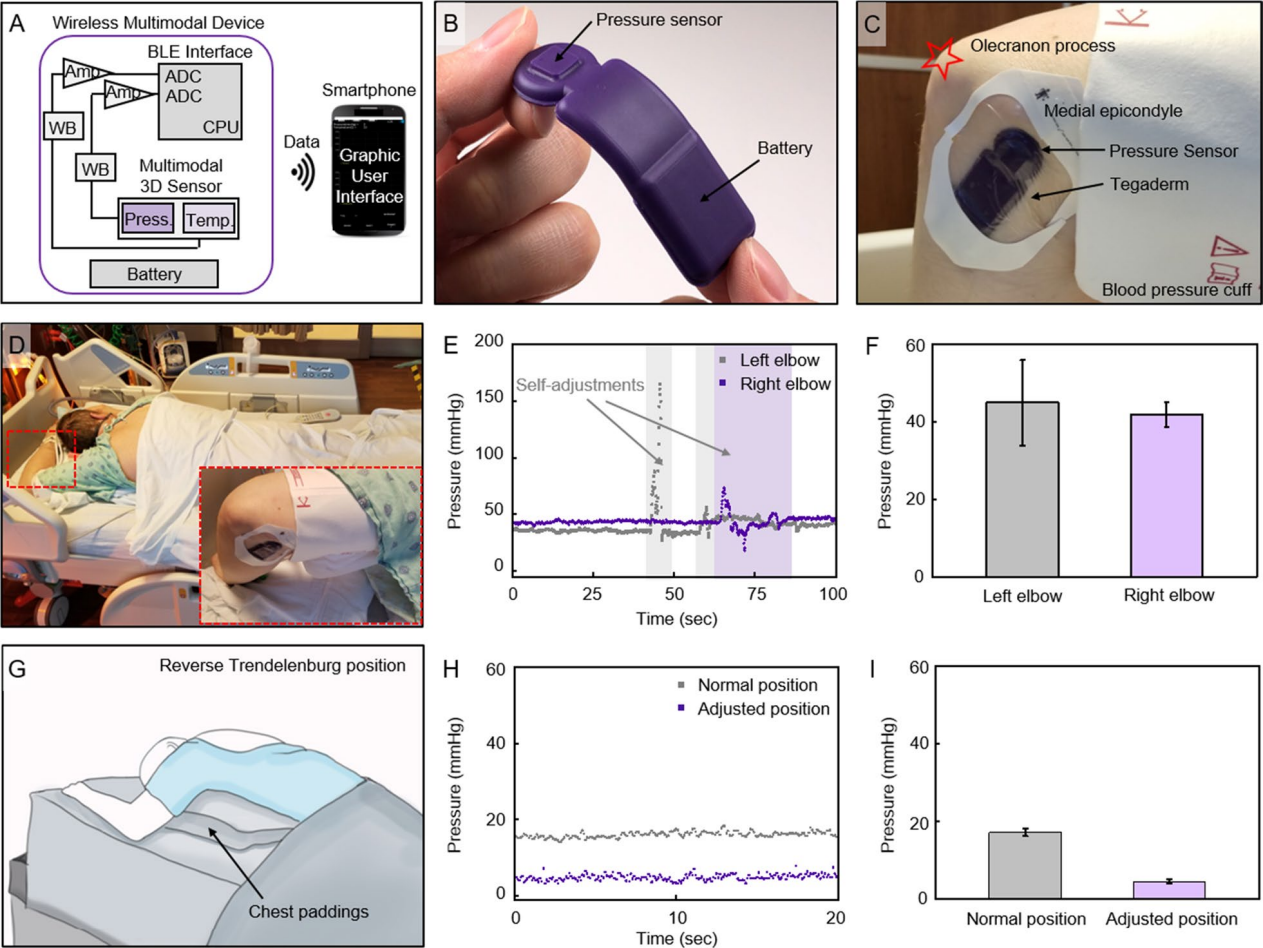
Soft, skin-interfaced sensor for wireless measurements of pressure for COVID19 patients in ICU. **A** Functional block diagram of the system that is powered through a battery, illustrating a Bluetooth Low Energy (BLE) system-on-a-chip (SoC), which connects to a Wheatstone bridge (WB) and an instrumentation amplifier (Amp) that convert and amplify the signal of the pressure sensor. The analog-to-digital converter (ADC)-sampled data passes through the central processing unit (CPU), which then transmits to BLE radio, displaying real-time data on the graphical user interface (smartphone). **B** Photograph of the sensor, depicting its thin and flexible form factor. **C** Photograph of a subject’s elbow, wearing the Tegaderm-secured sensor at the medial epicondyle. **G** Illustration of an intubated subject with the left arm up, in a reverse Trendelenburg position with chest paddings. **E** Representative pressure data of the self-proning subject at both left and right arms. Colored regions indicate self-adjustments. **F** Pressure measured in demonstrated normal prone position on both arms (measured at 10 Hz for 200, 300 s, respectively, data with individual sensor; error bar: SD). **D** Photograph of a self-proning subject (i.e. patient rolls themselves over on belly), placing his arms with the sensors upward. Red-dashed rectangle shows the unloading of the arm. **H** Representative pressure data of an intubated subject in the demonstrated normal prone position and the adjusted/optimized position. **I** Pressure measured in demonstrated normal prone position and adjusted, optimized prone position (measured at 10 Hz for 60, 900 s, respectively, data with the same sensor; error bar: SD)

In addition, proof of concept study of wireless, soft, skin-interfaced pressure sensor [15, 16] was performed on 2 patients admitted to ICU for severe COVID-19. This technology, comprised of a Bluetooth communication system, enables real-time, continuous, and wireless monitoring of pressure, through a smartphone (weight: ~ 4 g; dimensions: ~ 6.5 × 2 × 0.4 cm (length x width x height); Fig. 2A, B). After following a sterilization process, deployment of the sensor with a thin adhesive dressing (Tegaderm, 3 M) secured the intimate contact with the skin on the elbow (Fig. 2C). The sensor was applied adjacent to the ulnar nerve onto the medial epicondyle, without interfering with clinical standard-of-care equipment for the COVID-19 treatment (Fig. 2D). The wireless connection to a smartphone allowed for real-time visual display through a graphical user interface, data transmission, collection, and storage.

Statistics were descriptive in nature given the format of this study, retrospective case series and feasibility testing of a wearable, wireless sensor. Unless otherwise noted, values are expressed as mean ± standard deviation.

## Results

The baseline characteristics of 34 patients that acquired nerve injury after surviving severe COVID-19 are reported in Table 1. Of those patients, 4 are female (11.8%) and 30 are male (88.2%), with an average age of 53 ± 15 years (range 21–77) and average body mass index (BMI) of 33 ± 10 (range 17–59). Sixteen patients carry a diagnosis of diabetes mellitus (47%), and a total of 19 patients are classified as obese (BMI > 30; 56%). There are a total of 66 PNIs described here, which makes for an average of 1.9 ± 1.2 PNIs (range 1–6) per patient in our population. There are 12 different nerve injury sites in this case series, with the most common localizations being the common fibular (n = 16), ulnar (n = 12), sciatic (n = 9), radial (n = 6), and median (n = 6). Multifocal PNIs (≥ 2 sites) were noted in 53% (n = 18) of the cases. Electrodiagnostic testing was used to make the diagnosis for 57 of these PNIs (86.4%), and 53 of these 57 (93%) show electrophysiological evidence of more severe axonotmesis grade injury as compared to just 4 (7%) with a lesser neuropraxia (demyelinating) grade [17]. Imaging played an important role in the diagnosis and localization of a few cases. There were 5 cases (8%) of phrenic nerve injury that were diagnosed exclusively by ultrasound imaging, there were two cases of where the localization was refined from lumbar plexus to concurrent femoral and obturator nerve injuries (cases 4 and 11) on the basis of pelvic MRI, and 1 case of spinal accessory neuropathy confirmed by both MRI and electrodiagnostics (case 4). In just 3 out of 66 nerve injury sites (< 5%), the diagnosis was made on the basis of clinical exam by a neuromuscular specialist.

For the subset of these cases from the Shirley Ryan AbilityLab rehabilitation hospital (n = 22), as well as additional published cases from the same site (n = 12) [8], we calculated that these represented 10.7% (34 of 319) of the post-COVID-19 admissions over a 12 month period. All of the new cases from this site except for one (case #7; Table 1) had PNI diagnosed in the electrodiagnostic testing laboratory. In contrast, out of the non-COVID-19 related general medical rehabilitation admissions over the same period only 0.5% (3 of 572) had a PNI diagnosed in the electrodiagnostic testing laboratory. For the remaining admissions to this site, the majority of which included traumatic and non-traumatic disorders of the central nervous system, only 0.6% (13 of 2122) had a PNI diagnosed in the electrodiagnostic testing laboratory.

When our current data is combined with prior case series of PNIs in survivors of COVID-19 (Table 2), there are 183 PNIs, at 16 different anatomic sites in 92 patients. The most common localizations overall are ulnar (25.1%), common fibular (15.8%), sciatic (13.1%), median (9.8%), brachial plexus (8.7%) and radial nerve (8.2%), which is graphically summarized in Fig. 1. In several of the cases we report here we noted skin pressure sores overlaying the nerve compression sites including the fibular head and elbow. This was the inspiration for us to test the feasibility of wireless pressure sensor on COVID patients admitted to the ICU. Figure 1D illustrate the case of a patient being treated with high flow oxygen via nasal cannula and self proning for COVID-19 pneumonia. The subject was conscious during the protocol, which allowed for self-adjustments of the arms (Fig. 1E). The pressures from both left and right elbows at the medial epicondyle (Fig. 1E, F) show similar values, the averages of which are 44.9 mmHg and 41.7 mmHg, respectively. The inconsistency over time suggests that the subject repositioned the arms, which is also shown in the varying standard deviations, 11.0 mmHg and 3.2 mmHg, respectively. Figure 2G shows an illustration representing an intubated patient with respiratory failure from COVID-19 pneumonia, who was in a swimmer’s position and in a reverse Trendelenburg position with chest paddings. This variation of the prone position can be used to protect the ulnar nerve at the medial elbow [18] and is associated with the pressure reading to 4.5 ± 0.6 mmHg (Fig. 2H, I; purple). Gentle reloading of the medial elbow by the placement of a cushion underneath it increases the pressure to 17.2 ± 1.0 mmHg as detected by the wireless pressure sensor (Fig. 2H, I; grey).

## Discussion

We report on a large case series of 34 patients with 66 PNIs associated with survival from severe COVID-19. In majority of these cases there are ≥ 2 PNIs affecting the same person (Table 1). When taken in the context of our literature review of nerve injury case series associated with COVID-19 (n = 5–15 patients/study), plus the predominantly severe/axonal nerve injury characteristics (93% of current cases), these data imply that acquired PNIs are an important contributor to prolonged neurological impairments in survivors of COVID-19. The mechanisms underlying the propensity for PNI in COVID-19 critical illness is difficult to establish, but the anatomical distribution (Fig. 1) of these injuries implicate mechanical forces such as prolonged pressure against bony prominence leading to axonal injury from local ischemia [19]. As a potential strategy to address the risk of prolonged mechanical loading of peripheral nerves in the ICU, we demonstrate the feasibility of a wearable, wireless pressure sensor system to provide real time monitoring at the medial elbow (Fig. 2), which is most common site of compressive neuropathy in severe COVID-19 (Fig. 1).

The risk for focal PNIs in critically ill patients is well known [20], but the incidence has not been defined. In part this may be because these injuries may overlap with ICU-AW and not get the dedicated attention needed to diagnose with imaging [7] or electrodiagnostics [5]. In certain cases, like phrenic nerve injuries, the best diagnostic option may be a neuromuscular ultrasound study [21] but not all hospital systems have access to equipment and expertise for this diagnostic modality. Occasionally nerve compression in severe COVID-19 survivors may be accounted for by a hematoma [7] or an iatrogenic cause such as focal neuritis adjacent to central line site [21] that can be diagnosed by advanced imaging modalities.

Early results in severe COVID-19 survivors from single center case series put the incidence reported incidences between 14.5 and 16% [8, 9]. In the present report we report an incidence for a subset of the patients admitted to a single rehabilitation center of 10.7%, which is slightly lower than these prior reports. There were high rates of diabetes mellitus, obesity, male sex, and older age seen in our cohort which are characteristics of severe COVID-19-related ARDS patients [2], and risk factors for PNI in these patients [19, 22]. On a cellular level, a combination of inflammatory and immune-mediated injury caused by COVID-19 may increase susceptibility to nerve injury when patients have severe disease [23]. There is a paucity of evidence for direct SARS-CoV-2 of peripheral nerves, but this can’t be dismissed as a factor in a small subset of cases. [24]

Our use of wireless pressure sensors to monitor areas of ulnar nerve compression demonstrates a future approach to preventing prone positioning-related nerve injury. The sensors can provide real-time pressure information and can be used to adjust positioning at known compression sites before a compressive neuropathy occurs, while being used for extended periods of time (Fig. 2). Patients with severe COVID-19 appear particularly susceptible to positioning related PNIs [6, 7]. For example, the prone positioning intervention has been recommended for 12 to 16 h per day in mechanically ventilated adults with COVID-19 and refractory hypoxemia [25], but has associated with increase rates of acquired peripheral PNIs [6–8, 10–12]. Unfortunately, accurate details on ICU course were missing for the majority of patients in this case series since 27 out of 34 patients were diagnosed in free standing rehabilitation hospitals, rather than the original acute care hospitals so we did not attempt to associate PNIs with specific ICU positioning or protocols.

Other limitations of this study include lack of a control group, and the retrospective design, which precludes establishment of a causal relationship between patient positioning and peripheral nerve injury. Additionally, some patients with less severe PNIs may not have had advanced imaging or electrodiagnostic studies ordered, which may have led to a bias towards more severe PNIs and underestimation of PNI incidence.

Given the ongoing COVID-19 pandemic, and risks of new variants causing a resurgence of hospital admissions, further attention should be paid to the long-term sequela including PNIs. These injuries require long-term follow up and care with therapy and rehabilitation. Prevention and early identification of these injuries could help decrease additional morbidity of the disease. This study shows how the most common sites for nerve injury in severe COVID-19 patients map to known nerve entrapment sites vulnerable to mechanical loading (e.g. elbow, knee, ischium etc.), which highlights the handful of key anatomical locations that require extra attention to protect nerve health in the hospital setting as well as demonstrates the feasibility of a wearable, wireless pressure sensing system that can provide real time feedback in the ICU setting.

## Data Availability

will be made available on request**.**
